# Optimized Design and Radiation Error Correction of a Naturally Ventilated Air Temperature Sensor for Atmospheric Environmental Monitoring

**DOI:** 10.3390/s26123853

**Published:** 2026-06-17

**Authors:** Wei Jin, Qingquan Liu, Wei Dai, Xin Hong, Xilong Cao, Haiwen Sun

**Affiliations:** 1 Jiangsu Key Laboratory of Meteorological Observation and Information Processing, Nanjing University of Information Science and Technology, Nanjing 210044, China; 2 Jiangsu Collaborative Innovation Center on Atmospheric Environment and Equipment Technology, Nanjing University of Information Science and Technology, Nanjing 210044, China; 3 Key Laboratory of Micro-Electro-Mechanical System (MEMS) of the Ministry of Education, Southeast University, Nanjing 210096, China

**Keywords:** atmospheric environmental monitoring, temperature sensor, radiation error, computational fluid dynamics, neural network model

## Abstract

**Highlights:**

**What are the main findings?**

- A naturally ventilated air temperature sensor with symmetric guide plates and a dual aluminum-plate radiation shield was developed to reduce radiation error.
- CFD simulations identified a guide-plate spacing of 24 mm and an inclination angle of 45° as the preferred structural parameters.

**What are the implications of the main findings?**

- The MLP-based correction model achieved an RMSE of 0.052 °C and an MAE of 0.042 °C in field validation using a MET ONE Model 076B aspirated radiation shield (Met One Instruments, Inc., Grants Pass, OR, USA) as the reference.
- The proposed sensor provides a low-power and easy-to-maintain solution for air temperature monitoring in meteorological, air-quality, and agricultural microclimate applications.

**Abstract:**

Air temperature measurements in atmospheric environmental monitoring are susceptible to radiation-induced bias under natural ventilation. This study develops a low-power naturally ventilated air temperature sensor and a correction method combining computational fluid dynamics (CFD) with machine learning. The sensor integrates a Pt100 thin-film platinum resistance probe (Heraeus Holding GmbH, Hanau, Germany), symmetric guide plates, and a dual aluminum-plate radiation shield to reduce radiative heating while improving airflow around the probe. A three-dimensional fluid–solid coupled heat-transfer model was established in ANSYS FLUENT 15.0 to optimize guide-plate spacing and inclination angle and quantify the effects of solar radiation, long-wave radiation, scattered radiation, air density, wind speed, solar elevation angle, and surface albedo on radiation error. CFD results identified a guide-plate spacing of 24 mm and an inclination angle of 45° as the preferred parameters. A multilayer perceptron (MLP) model trained with CFD-derived data was validated in field experiments using a Model 076B aspirated radiation shield (Met One Instruments, Inc., Grants Pass, OR, USA) as the reference. The model predicted radiation error with a root mean square error (RMSE) of 0.052 °C, a mean absolute error (MAE) of 0.042 °C, and a correlation coefficient of 0.92. The proposed sensor and correction method provide a low-power and easy-to-maintain approach for reducing radiation-induced bias in naturally ventilated air-temperature measurements, with potential applications in meteorological observation, air-quality monitoring, and agricultural microclimate assessment.

## 1. Introduction

Atmospheric environmental monitoring is a fundamental component of meteorological research, pollution prevention and control, and climate change assessment. Among various meteorological parameters, accurate air temperature observation plays a critical role in weather forecasting, environmental quality assessment, and long-term climate trend analysis [1,2,3]. High-precision temperature observations not only provide reliable data support for the development of meteorological and environmental models but also serve as an important basis for evaluating regional environmental changes and formulating corresponding response strategies [4,5]. According to the sixth report released by the Intergovernmental Panel on Climate Change (IPCC), global temperature rise will substantially increase risks to ecosystems and human society [6]. Therefore, improving the accuracy of air temperature measurements is of considerable practical significance, particularly as environmental monitoring and climate-related applications impose increasingly stringent requirements on atmospheric temperature observation accuracy and data reliability.

Radiation error is one of the major factors affecting the accuracy of air temperature measurements. Direct solar radiation, terrestrial long-wave radiation, and atmospheric diffuse radiation can heat the temperature-sensing element and its surrounding support structures, causing the measured temperature to deviate from the true free-air temperature [7,8,9]. Conventional Stevenson screens and naturally ventilated radiation shields can reduce direct radiation interference to some extent; however, considerable measurement errors may still occur under unfavorable observational conditions, such as intense solar irradiance and weak wind [10,11,12]. Previous studies have reported that the radiation error of conventional naturally ventilated shielding structures can exceed 0.5 °C and may even be greater than 1 °C under certain observation conditions [13,14]. To reduce such errors, various improved radiation protection devices have been developed. For example, the Model 43502 aspirated radiation shield produced by R.M. Young can limit temperature errors to approximately 0.2 °C under relatively high forced ventilation velocities [15]. Thomas et al. proposed a double-tube aspirated radiation shield that can control temperature errors within ±0.08 °C [16]. Nevertheless, aspirated radiation shields rely on active ventilation and may encounter problems such as energy consumption, dust accumulation, icing, insect blockage, and maintenance costs during long-term field deployment, which limits their broader application in distributed environmental monitoring.

To overcome these limitations, this study proposes a naturally ventilated air temperature sensor for atmospheric environmental monitoring and establishes a radiation error correction method combining CFD simulations and machine learning. First, a naturally ventilated sensor structure with both radiation shielding and airflow-guiding functions is designed to simultaneously reduce external radiation effects and enhance ventilation in the probe region. Second, a CFD-based fluid–solid coupled heat transfer model [17,18,19] is employed to perform parametric optimization of the guide-plate spacing and inclination angle, and the effects of multiple environmental factors on radiation error are analyzed. Third, a radiation error dataset under multiple environmental conditions is constructed, the prediction performance of different regression models is compared, and a radiation error correction model is established. Finally, field comparison experiments are carried out using a MET ONE Model 076B aspirated radiation shield as the reference instrument to assess the performance of the proposed sensor structure and correction method.

## 2. Temperature Sensor Structural Design and CFD Modeling

### 2.1. Structural Design of the Naturally Ventilated Temperature Sensor

**Figure 1:**
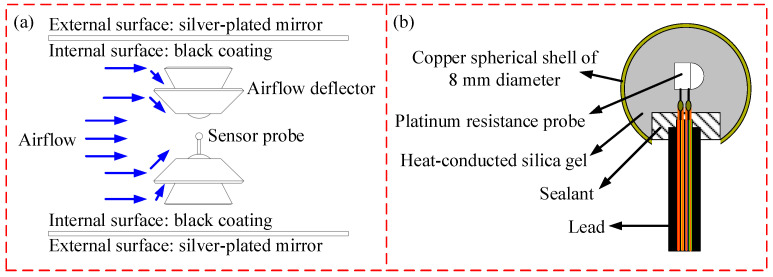
Structural illustration of the proposed sensor: (**a**) overall sensor configuration; (**b**) probe design.

The developed naturally ventilated air temperature sensor mainly consists of a sensing probe, a copper spherical shell, symmetric guide plates, upper and lower aluminum plates, and a supporting structure, as shown in Figure 1. The sensing probe uses a Pt100 thin-film platinum resistance element as the temperature-sensitive component and is mounted centrally inside an 8 mm-diameter copper spherical shell. Owing to its high thermal conductivity, the copper spherical shell enhances heat exchange between the sensing probe and the surrounding air, thereby improving the probe response to ambient air temperature variations. The openings of the spherical shell are sealed with waterproof adhesive, and the interior is filled with thermal grease to improve heat transfer efficiency.

The symmetric guide plates are key components of the proposed sensor structure. They are made of plastic and coated with a white surface layer to reduce solar radiation absorption. Their primary function is to alter the flow direction of natural wind entering the radiation shield, allowing more airflow to pass through the region near the sensing probe. This enhances air exchange around the probe and reduces localized heat accumulation.

The upper and lower aluminum plates are installed on the two sides of the sensing probe in the vertical direction, respectively, and serve as the main radiation shielding components of the proposed sensor. The outer surfaces of the aluminum plates are coated with a highly reflective silver coating to reflect direct solar radiation and surface-reflected short-wave radiation. The inner surfaces are coated with a black layer having an absorptivity of 0.9 to absorb secondary reflected radiation entering the internal structure, thereby reducing the influence of multiple reflections on the probe. The upper aluminum plate mainly attenuates solar short-wave radiation, whereas the lower aluminum plate primarily reduces terrestrial long-wave radiation and reflected radiation.

### 2.2. CFD Simulation Model and Boundary Conditions

**Figure 2:**
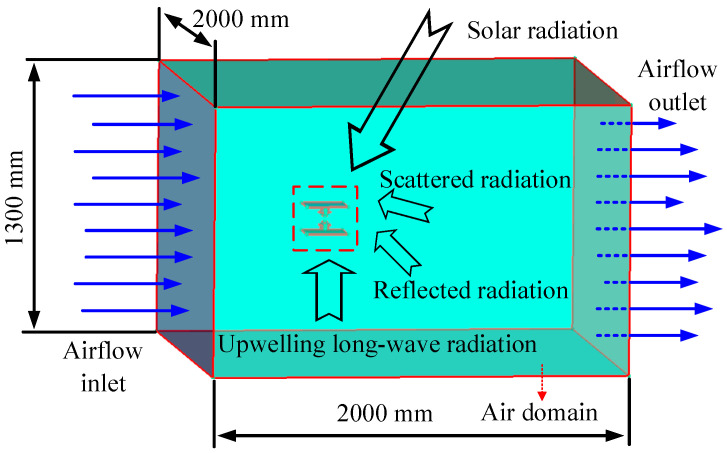
Schematic of the CFD computational domain and main boundary conditions.

To analyze the internal flow-field distribution of the sensor and the radiation-induced temperature rise of the sensing probe, the three-dimensional model was meshed using ANSYS ICEM CFD 15.0, and a conjugate heat transfer model was established in ANSYS FLUENT. The computational domain consisted of the solid structure of the temperature sensor and the surrounding air domain, as shown in Figure 2. A velocity-inlet condition was assigned to the inlet boundary, whereas a pressure-outlet condition was applied at the outlet boundary. The remaining external boundaries were treated as no-slip walls. Coupled heat transfer interfaces were assigned between the solid components and the air domain to describe heat exchange among the incoming airflow, the sensor structure, and the sensing probe. Table 1 summarizes the thermophysical parameters assigned to each material in the CFD model.

To ensure the numerical reliability of the CFD results, a mesh independence test was conducted before the parametric simulations. Three mesh levels were compared using the sensing-probe temperature as the monitoring variable, and the results are shown in Table 2. The probe temperature changed from 300.094 K to 300.092 K as the mesh was refined from the coarse to the medium level, while further refinement to the fine level produced no noticeable change. Therefore, the medium mesh was adopted in subsequent simulations to balance accuracy and computational cost. During the iterative solution, all residuals were monitored, and the energy residual was reduced below 10^−6^. The sensing-probe temperature was also monitored to ensure that a stable solution was obtained.

## 3. Parametric Optimization of the Temperature Sensor Structure

### 3.1. Parametric Simulation Settings

To optimize the structural parameters of the sensor, parametric simulations were conducted using the single-factor control method. During structural optimization, the sensing probe temperature and the local airflow velocity around the probe were selected as the primary evaluation metrics. The probe temperature was used to characterize the local temperature rise caused by radiation, whereas the local airflow velocity was used to evaluate the natural ventilation capability. The structural parameters of the sensor were optimized using CFD-based single-factor parametric screening rather than a global optimization algorithm. The objective was to minimize the radiation-induced temperature rise of the sensing probe while maintaining adequate local airflow. Accordingly, the probe temperature was used as the optimization metric, and the local airflow velocity was used as the ventilation constraint. The final parameters were selected by comparing these two indicators under identical simulation conditions.

In the simulations, the energy equation was enabled, the standard k–ε turbulence model was adopted for airflow modeling, standard wall functions were applied for near-wall treatment, and pressure–velocity coupling was handled using the SIMPLE algorithm [20,21]. Solar radiation was applied using the solar-ray-tracing model, while direct solar radiation, scattered radiation, upward long-wave radiation, convective heat transfer with air, and heat conduction within solids were considered simultaneously. The baseline operating conditions were set as follows: ambient air temperature of 300 K, inlet wind speed of 1 m/s, solar radiation intensity of 1000 W/m^2^, scattered radiation intensity of 200 W/m^2^, long-wave radiation intensity of 300 W/m^2^, air density of 1.225 kg/m^3^, solar elevation angle of 45°, surface albedo of 0.2, and gravitational acceleration of 9.8 m/s^2^. In each parametric case, all material parameters, boundary conditions, and solver settings were kept unchanged except for the structural parameter under investigation.

### 3.2. Parametric Optimization of Guide-Plate Spacing

Guide-plate spacing is an important structural parameter affecting the internal ventilation capacity of the sensor and heat diffusion in the probe region. If the spacing between the upper and lower guide plates is too small, shielding against oblique and scattered radiation may be enhanced, but airflow entering the region near the probe may be restricted, resulting in insufficient air exchange around the sensing probe. Conversely, excessive spacing can enlarge the airflow passage and facilitate natural ventilation but may weaken the local radiation shielding effect. Therefore, the guide-plate spacing requires a balance between radiation shielding performance and natural ventilation capability.

**Figure 3:**
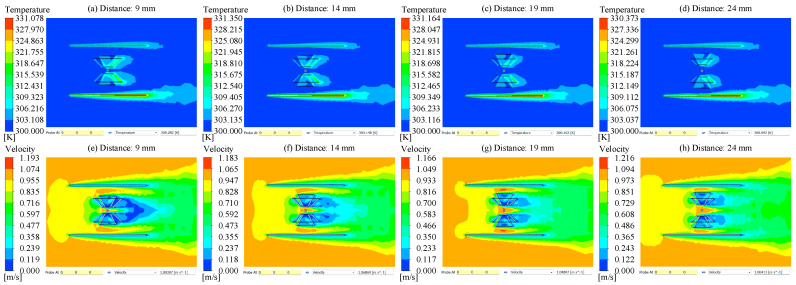
Temperature and velocity distributions of the proposed sensor under different guide-plate spacings. Panels (**a**–**d**) show the temperature-field distributions for spacings of 9, 14, 19, and 24 mm, respectively; panels (**e**–**h**) show the corresponding velocity-field distributions.

To investigate the influence of guide-plate spacing on temperature-field and velocity-field distributions, four spacing schemes were considered: 9, 14, 19, and 24 mm. The temperature and velocity distributions of the proposed sensor under different spacing conditions are presented in Figure 3.

The results show that the sensing probe temperature gradually decreases as the guide-plate spacing increases, with values of 300.282 K at 9 mm, 300.148 K at 14 mm, 300.102 K at 19 mm, and 300.092 K at 24 mm. Meanwhile, the airflow velocities around the probe are 1.08387, 1.06869, 1.04897, and 1.06411 m/s, respectively. Although the local airflow velocity fluctuates slightly with spacing, it generally remains within the natural ventilation range. Considering both the suppression of radiation-induced temperature rise and ventilation performance, the 24 mm spacing scheme performed better in reducing the probe temperature rise caused by radiation.

### 3.3. Parametric Optimization of Guide-Plate Inclination

**Figure 4:**
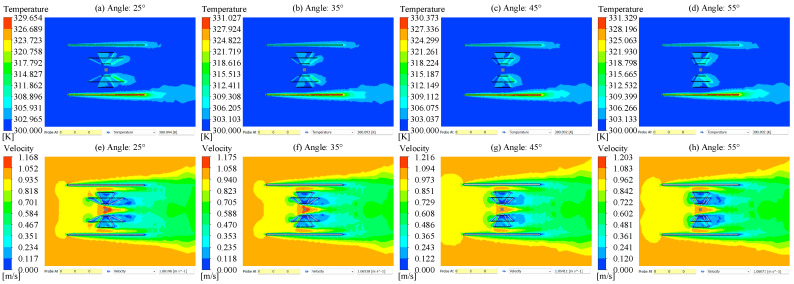
Temperature and velocity distributions of the proposed sensor under different guide-plate inclination angles. Panels (**a**–**d**) show the temperature-field distributions for inclination angles of 25°, 35°, 45°, and 55°, respectively; panels (**e**–**h**) show the corresponding velocity-field distributions.

Based on the optimized guide-plate spacing obtained above, the guide-plate inclination angle was further investigated. The guide-plate spacing was fixed at 24 mm, while all other structural dimensions, material parameters, and boundary conditions remained unchanged. Only the inclination angle of the upper and lower symmetric guide plates was varied. The inclination angle was measured from the horizontal plane to the inclined surface of the guide plate. Four inclination angles, namely 25°, 35°, 45°, and 55°, were considered. The temperature and velocity distributions of the sensor under different inclination angles are shown in Figure 4.

The simulation results indicate that the sensing probe temperature decreases slightly with increasing guide-plate inclination angle, with values of 300.094 K at 25°, 300.093 K at 35°, 300.092 K at 45°, and 300.092 K at 55°. The corresponding airflow velocities around the probe are 1.06196, 1.06538, 1.06411, and 1.06071 m/s, respectively. These results suggest that the probe temperature varies only slightly among different inclination angles, whereas the airflow distribution differs among the cases. When the inclination angle is too small, the airflow-guiding effect is insufficient, making it difficult for airflow to fully enter the probe region. When the inclination angle is too large, local flow resistance may increase and low-velocity regions may form inside the sensor. Considering both temperature-rise suppression and local ventilation, 45° can be selected as the preferred guide-plate inclination angle for the proposed structure.

### 3.4. Influence of Environmental Factors Under the Optimized Structure

**Figure 5:**
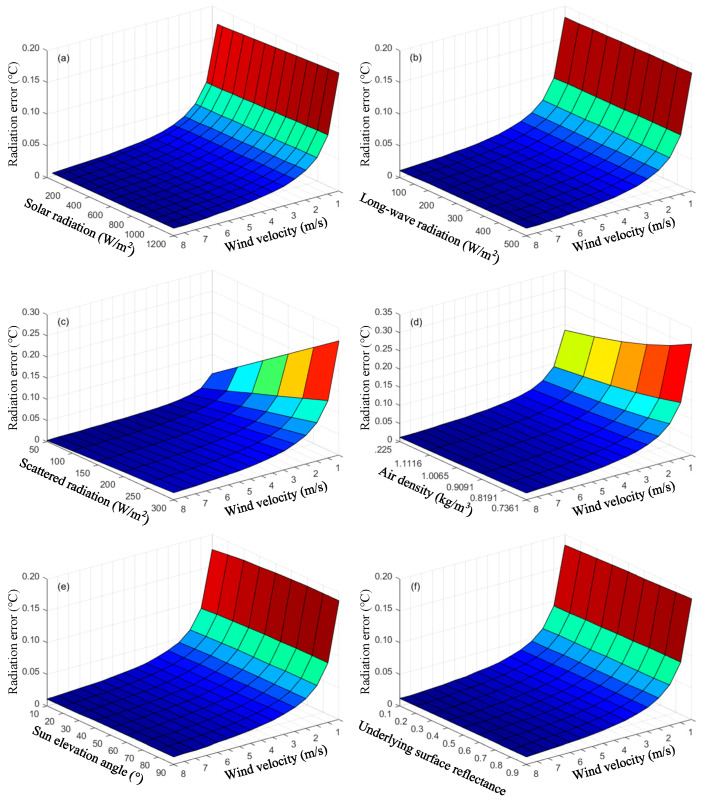
Radiation error of the proposed sensor under varying environmental scenarios: (**a**) solar radiation; (**b**) long-wave radiation; (**c**) scattered radiation; (**d**) air density; (**e**) solar elevation angle; (**f**) surface albedo.

After optimizing the guide-plate spacing and inclination angle, the effects of different environmental conditions on the radiation error Δ*T* were further evaluated. Specifically, the influences of solar radiation *P*_1_, long-wave radiation *P*_2_, scattered radiation *P*_3_, air density *ρ*, ambient wind speed *V*, solar elevation angle *E*, and surface albedo *f* on radiation error were analyzed. Table 3 provides the variation ranges of these variables, and Figure 5 presents the corresponding simulation results.

The results indicate that the radiation error increases with increasing *P*_1_, *P*_2_, and *P*_3_. The upper aluminum plate attenuates most of the direct *P*_1_, whereas the lower aluminum plate primarily reduces the effects of *P*_2_ and radiation reflected from the underlying surface. As *P*_1_ increases from 50 to 1200 W/m^2^, Δ*T* changes from 0.088 to 0.093 °C, as shown in Figure 5a. When *P*_2_ increases from 50 to 500 W/m^2^, Δ*T* varies within the range of 0.091–0.093 °C, as shown in Figure 5b. Since *P*_3_ mainly originates from the scattering of solar radiation by gas molecules, clouds, and aerosols in the atmosphere, it is difficult to completely shield this component structurally. Therefore, *P*_3_ has a more pronounced influence on the radiation error. As *P*_3_ increases from 50 to 300 W/m^2^, Δ*T* increases from 0.028 to 0.136 °C, as shown in Figure 5c. With decreasing air density, the convective heat transfer capacity of air weakens, resulting in a significant increase in radiation error. When *ρ* decreases from 1.225 to 0.7361 kg/m^3^, Δ*T* increases from 0.092 to 0.153 °C, as shown in Figure 5d. When *E* varies from 10° to 90°, Δ*T* ranges from 0.088 to 0.094 °C, as shown in Figure 5e. When the surface albedo *f* varies between 0.1 and 0.9, Δ*T* changes from 0.092 to 0.095 °C, as shown in Figure 5f.

## 4. Construction of the Radiation Error Correction Model

### 4.1. Dataset Construction and Preprocessing

To establish a radiation error correction model applicable to different environmental conditions, a sample dataset was constructed based on the CFD simulation results of the optimized structure. The input variables of the model were *P*_1_, *P*_2_, *P*_3_, *ρ*, *V*, *E*, and *f*, while the output variable was the Δ*T*. The input vector can be expressed as:

(1) $$x={\left(P_{1},P_{2},P_{3},\rho ,V,E,f\right)}^{T}$$

Because the input variables have different units and value ranges, Z-score normalization [22] was applied to the input features to improve the convergence stability of model training:

(2) $$x_{ij}^{*}=\frac{x_{ij}-\mu_{j}}{\sigma_{j}}$$

where $x_{ij}$ denotes the raw value of the *j*-th input variable for the *i*-th sample; $\mu_{j}$ and $\sigma_{j}$ denote the mean and standard deviation of the corresponding input variable in the training set, respectively; and $x_{ij}^{*}$ is the normalized input variable. The normalization parameters were derived exclusively from the training set and subsequently used to transform the validation set, test set, and field prediction data to ensure consistent transformation. The output variable Δ*T* was also normalized, and the model predictions were inverse-transformed to the original temperature scale for subsequent error evaluation.

### 4.2. Neural Network Architecture

Radiation error is jointly affected by *P*_1_, *P*_2_, *P*_3_, *ρ*, *V*, *E*, and *f*, and there are obvious nonlinear relationships between these variables and the radiation error. Conventional linear models are insufficient to fully describe such complex mapping relationships. Therefore, an MLP neural network was adopted to construct the radiation error correction model.

**Figure 6:**
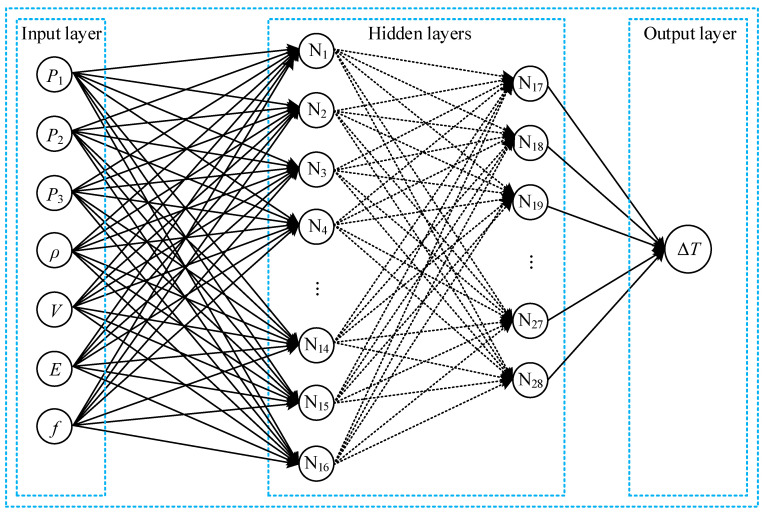
Architecture of the MLP model.

The developed MLP model is organized with one input layer, two hidden layers, and one output layer. Seven neurons are assigned to the input layer to represent *P*_1_, *P*_2_, *P*_3_, *ρ*, *V*, *E*, and *f*. The output layer contains one neuron corresponding to the predicted radiation error Δ*T*_pred_. To enhance the ability of the model to capture complex nonlinear relationships, a two-hidden-layer architecture was employed. The two hidden layers comprise 16 and 12 neurons, respectively, resulting in a “7–16–12–1” network topology, as shown in Figure 6.

The activation function adopted for the hidden-layer neurons was the rectified linear unit (ReLU), which is expressed as:

(3) ReLU=x,x>00,x≤0

The forward propagation process of the neural network can be expressed as:

(4) A1=ReLUW1X+b1

(5) A2=ReLUW2A1+b2

(6) ΔT=ReLUW3A2+b3

where *X* is the normalized input matrix; *W*[1], *W*[2], and *W*[3] denote the weight matrices associated with the input-to-hidden, hidden-to-hidden, and hidden-to-output transformations, respectively; *b*[1], *b*[2], and *b*[3] denote the corresponding bias terms; and *A*[1] and *A*[2] represent the outputs generated by the two hidden layers.

### 4.3. Multi-Model Comparison and Model Selection

To evaluate the predictive capability of different regression models for radiation error, multiple linear regression (MLR), support vector regression (SVR), and MLP were selected for comparative analysis [23,24,25]. MLR was used as a linear baseline model, SVR was employed to examine the applicability of kernel-based methods to nonlinear regression problems, and MLP was used to fit the complex nonlinear mapping between multiple environmental factors and radiation error.

**Figure 7:**
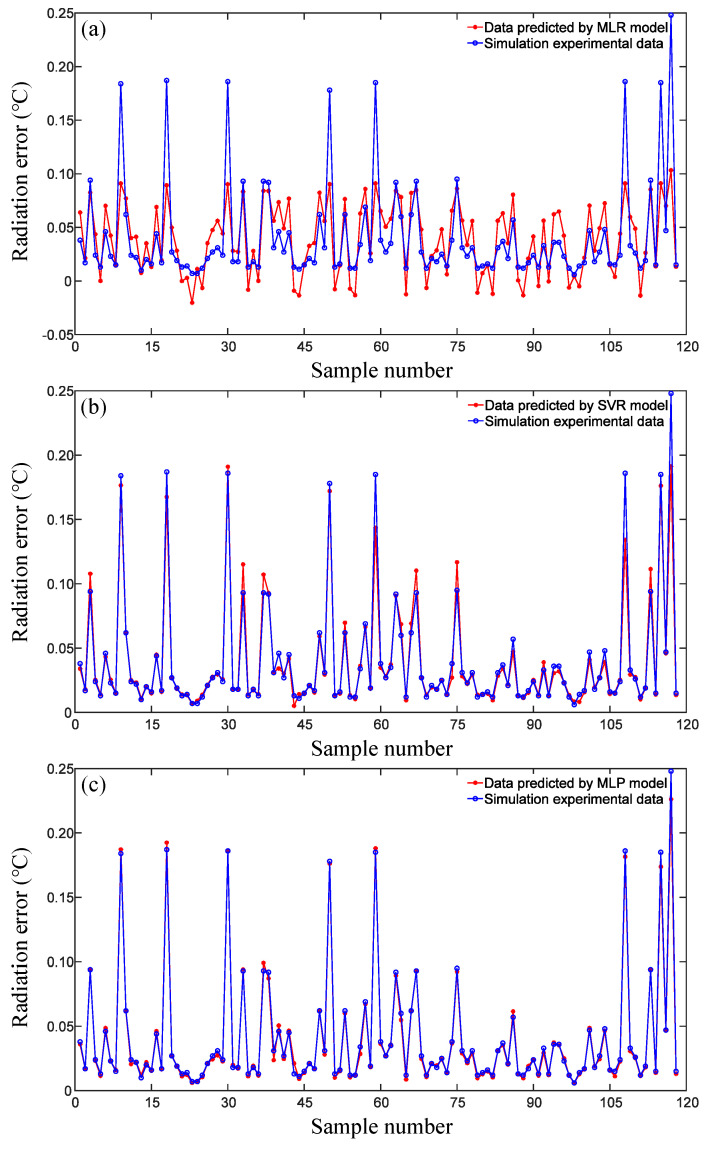
Comparison between radiation error predictions of different models and CFD simulation results: (**a**) MLR model; (**b**) SVR model; (**c**) MLP model.

To ensure fairness in model comparison, MLR, SVR, and MLP were compared using the same input variables and the same training and test datasets. The model inputs were the normalized variables *P*_1_, *P*_2_, *P*_3_, *ρ*, *V*, *E*, and *f*, and the output was the predicted radiation error Δ*T*_pred_. A total of 784 samples were allocated to the training, validation, and test sets in proportions of approximately 70%, 15%, and 15%, respectively. The validation set was used for early stopping during MLP training. The CFD-based test set was used only for internal model comparison, while the independent field-validation dataset in Section 5.3 was used for external evaluation under real outdoor conditions. For the MLP model, the input features were standardized using the mean and standard deviation of the training set, and the same transformation was applied to the validation, test, and field-validation data. The output variable Δ*T* was also standardized during training and inverse-transformed for performance evaluation. The model was trained using the Adam optimizer with mean squared error as the loss function, an initial learning rate of 0.001, a batch size of 8, and a maximum of 1000 epochs. Early stopping was applied based on the validation loss with a patience of 30 epochs, and the best model weights were restored. To improve reproducibility, the random seed was fixed at 0 for Python 3.10.0, NumPy 1.24.1, and TensorFlow 2.11.0. The test samples were then fed into the three models and compared with the CFD-simulated values, as shown in Figure 7.

To quantitatively evaluate the prediction performance of the three models in radiation error correction, RMSE, MAE, and the correlation coefficient r were selected as evaluation metrics. RMSE characterizes the overall dispersion of model prediction errors, MAE indicates the mean absolute discrepancy between predicted and simulated values, and r evaluates the consistency between model predictions and CFD simulation results. These metrics are calculated as follows:

(7) $$\mathrm{RMSE}=\sqrt{\frac{\sum_{i=1}^{n}{\left(\Delta T_{i}-\Delta T_{\mathrm{pred},i}\right)}^{2}}{n}}$$

(8) $$\mathrm{MAE}=\frac{1}{n}\sum_{i=1}^{n}\left|\Delta T_{i}-\left.\Delta T_{\mathrm{pred},i}\right|\right.$$

(9) $$r=\frac{\sum_{i=1}^{n}\left(\Delta T_{i}-\bar{\Delta T}\right)\cdot \left(\Delta T_{\mathrm{pred},i}-{\bar{\Delta T}}_{\mathrm{pred}}\right)}{\sqrt{{\sum_{i=1}^{n}{\left(\Delta T_{i}-\bar{\Delta T}\right)}^{2}\cdot \sum_{i=1}^{n}\left(\Delta T_{\mathrm{pred},i}-{\bar{\Delta T}}_{\mathrm{pred}}\right)}^{2}}}$$

where Δ*T_i_* denotes the CFD-simulated radiation error of the *i*-th sample, Δ*T*_pred,*i*_ denotes the model-estimated radiation error for sample *i*, *n* represents the test-set sample size, and $\bar{\Delta T}$ and ${\bar{\Delta T}}_{\mathrm{pred}}$ are the mean values of the CFD-simulated and predicted radiation errors, respectively. The evaluation results of the three models on the test set are listed in Table 4.

As shown in Table 4, the MLR model shows relatively large RMSE and MAE values and a relatively low correlation coefficient, indicating that a linear model cannot adequately capture the nonlinear relationship between multiple environmental factors and radiation error. The SVR model achieves substantially higher prediction accuracy than MLR, suggesting that the radiation error correction problem exhibits strong nonlinear characteristics. In comparison, the MLP model achieves the lowest RMSE and MAE on the test set, with a correlation coefficient of 0.9979, indicating high consistency between the predicted values and CFD-simulated values. Therefore, considering both test-set prediction accuracy and nonlinear fitting capability, the MLP model was ultimately selected as the radiation-error compensation approach for the naturally ventilated temperature sensor.

## 5. Field Validation and Performance Analysis

### 5.1. Construction of the Radiation Observation System

**Figure 8:**
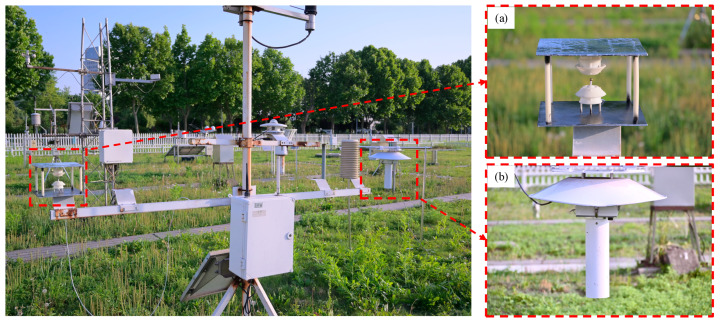
Radiation error observation platform: (**a**) proposed sensor; (**b**) 076B aspirated radiation shield.

To validate the measurement performance of the proposed temperature sensor and the effectiveness of the radiation error correction model, a field comparative observation platform was constructed at the Nanjing Meteorological Observatory under the China Meteorological Administration, located at 32.12° N and 118.42° E with an altitude of 22 m, as shown in Figure 8. A Pt100 element was installed inside the proposed sensor as the sensing element. To improve the reliability of temperature measurements, the Pt100 was calibrated before field deployment.

In the field comparative experiment, a MET ONE Model 076B aspirated radiation shield was used as the reference instrument [26]. This instrument employs aspirated ventilation and is widely used as a high-precision reference device in temperature observations involving radiation error. During the experiment, the proposed temperature sensor and the 076B radiation shield were installed at nearby positions and exposed to as similar environmental exposure conditions as possible to minimize the influence of installation-location differences on the observations.

In addition to temperature measurements, relevant environmental parameters were recorded synchronously. Solar radiation was recorded with a CMP-21 pyranometer from Kipp & Zonen, Delft, The Netherlands, and wind speed was measured using a 03002V anemometer (R.M. Young Company, Traverse City, MI, USA). The output signals from the temperature probe, pyranometer, and anemometer were collected by the same data acquisition system to ensure temporal synchronization throughout the experiment.

### 5.2. Measurement of Environmental Parameters

During the experiment, solar radiation and wind speed were measured using the aforementioned CMP-21 pyranometer and 03002V anemometer, respectively. Considering that the albedo of grass-covered surfaces is generally within the range of 0.15–0.25, the surface albedo was set to 0.2 in this study [27]. Long-wave radiation was calculated as follows [28]:

(10) $$I_{l,0}^{↑}=0.95\sigma T_{g}^{4}$$

where *T_g_* denotes the ground surface temperature, and *σ* is the Stefan–Boltzmann constant, with *σ* = 5.67 × 10^−8^ W·m^−2^·K^−4^.

In addition to the directly measured solar radiation and wind speed, the remaining input variables required by the MLP correction model were obtained through calculation or empirical approximation. *P*_3_ was empirically estimated according to the measured solar radiation level and observed weather conditions, *E* was calculated from the geographic coordinates of the experimental site, observation date, and local time, and *ρ* was approximated as 1.225 kg/m^3^ considering the low altitude of the experimental site. The surface albedo was set to 0.2 based on the typical albedo range of grass-covered surfaces.

**Figure 9:**
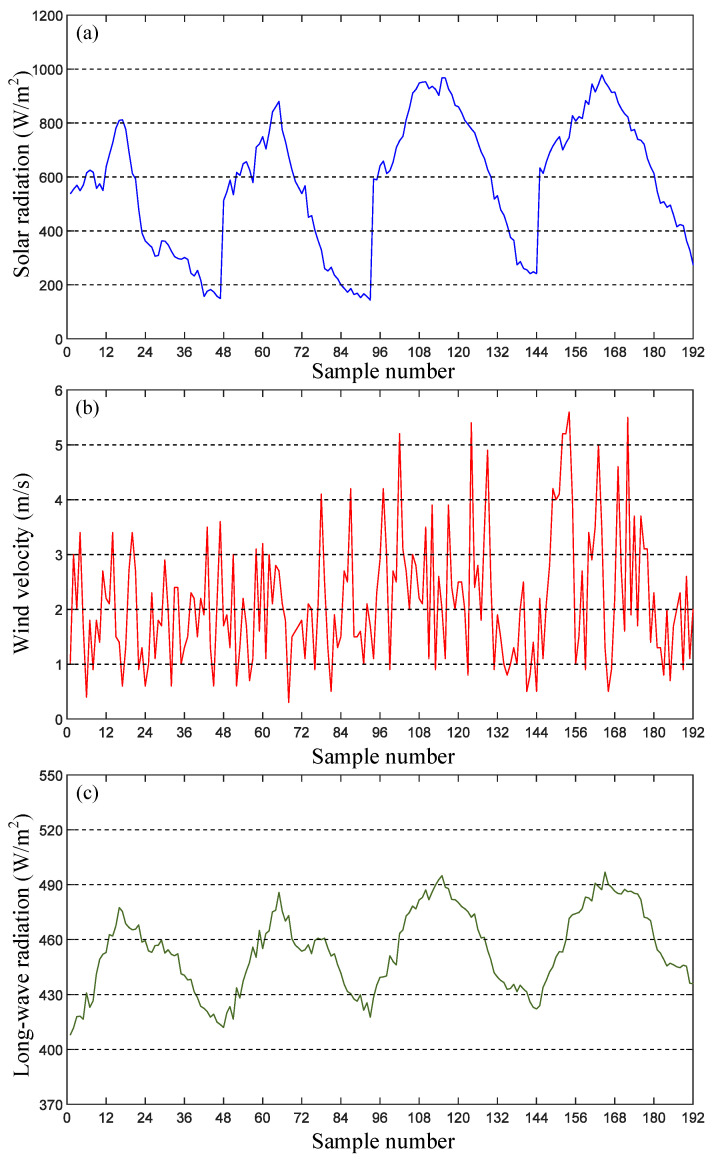
Measured results of *P*_1_, *V*, and *P*_2_: panels (**a**–**c**) correspond to the three parameters, respectively.

Based on the above instruments and calculation method, solar radiation intensity, wind speed, and long-wave radiation were synchronously obtained during the field experiment. All variables were recorded at 10 min intervals with consistent timestamps to ensure matching between environmental parameters and corresponding temperature observations. The relevant data collected during the three-day field experiment are shown in Figure 9.

### 5.3. Radiation Error Analysis

**Figure 10:**
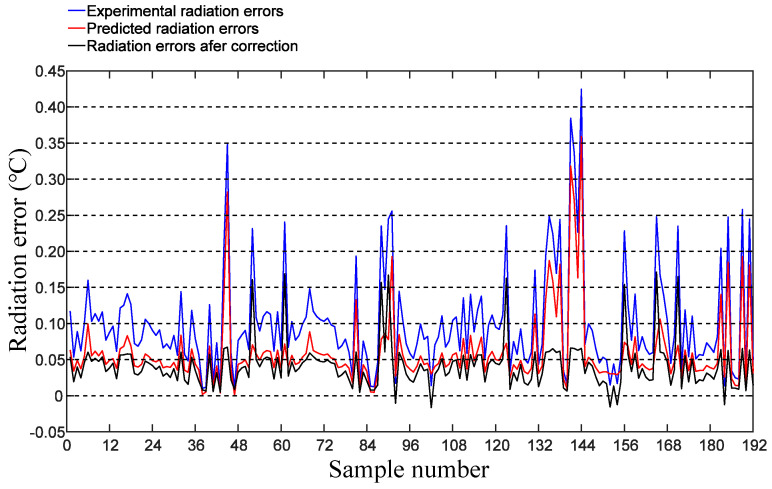
Comparative analysis of experimental radiation error, predicted radiation error, and corrected radiation error.

The temperature measured by the 076B was used as the reference temperature. The experimental radiation error was defined as the temperature offset of the proposed sensor relative to the reference temperature. The environmental parameters shown in Figure 9 were introduced into the MLP model to estimate the predicted radiation error. The corrected radiation error was then calculated as the temperature offset of the corrected sensor output relative to the reference temperature. Figure 10 presents the comparative results for the experimental radiation error, predicted radiation error, and corrected radiation error.

The comparison in Figure 10 provides an experimental evaluation of the CFD-trained MLP correction framework under independent field conditions. The field-validation dataset was not used for model training, validation, or testing, but served as an independent external evaluation dataset. The results show that the MLP model exhibits good predictive capability for radiation error on the independent field test dataset, with an RMSE of 0.052 °C, an MAE of 0.042 °C, and a correlation coefficient of 0.92. Compared with the uncorrected results, the corrected radiation error is significantly reduced overall, indicating that the established correction model can effectively improve the performance of air temperature measurements under natural ventilation conditions.

To further quantify the correction performance, error statistics were calculated using the same field-validation dataset. The uncorrected error was defined as the temperature difference between the proposed sensor and the 076B reference, while the corrected error was defined as the temperature difference between the corrected sensor output and the reference. The MBE, RMSE, MAE, maximum absolute error, and 95th percentile absolute error are summarized in Table 5.

Table 5 shows that the MLP-based correction reduced all error metrics in the field-validation dataset, demonstrating improved accuracy and stability of the corrected temperature measurements under field conditions. Compared with conventional naturally ventilated shields, for which radiation errors may exceed 0.5 °C or even 1 °C under strong radiation and weak wind conditions, the proposed sensor achieved a corrected RMSE of 0.052 °C and an MAE of 0.042 °C in the field validation, while avoiding the power consumption and maintenance requirements associated with aspirated shields.

The empirical estimation of *P*_3_, the fixed surface albedo, and the approximation of *ρ* may introduce uncertainties associated with sky conditions, surface states, atmospheric pressure, and humidity. These factors may partly explain the difference between the CFD test-set accuracy and the field-validation accuracy and will be further addressed through more complete radiation and meteorological measurements in future work. Although the present study focuses on radiation-induced errors, other potential error sources, including sensor self-heating, conductive heat transfer through supporting components and lead wires, and humidity-related effects, were not independently quantified. These factors will be further investigated in future long-term experiments. More generally, the field validation was limited to a single site and a relatively narrow range of outdoor conditions. Therefore, the results should be interpreted as a preliminary evaluation of the proposed sensor and correction framework. Further long-term and multi-site experiments covering different land-cover surfaces, terrains, altitudes, climatic regions, seasons, and exposure conditions are needed to assess its robustness and generalization capability.

## 6. Conclusions

To meet the demand for high-accuracy, low-power-potential air temperature measurement in atmospheric environmental monitoring, the present work designed a natural-ventilation air temperature sensor and established a radiation error correction method combining CFD simulations with an MLP neural network. The effectiveness of the proposed sensor structure and error correction method was verified through numerical simulation, structural parameter optimization, model comparison, and field comparative experiments. The key findings are summarized as follows:

(1) The developed temperature sensor consists mainly of a Pt100 sensing probe, a pair of symmetric guide plates, and a dual-aluminum-plate radiation shielding structure. This structure can mitigate the influence of direct solar radiation, terrestrial long-wave radiation, and reflected radiation while guiding natural airflow through the sensing probe region, thereby improving air exchange conditions near the probe.
(2) Guide-plate spacing markedly affects the internal temperature and velocity fields of the sensor. As the guide-plate spacing increases from 9 to 24 mm, the sensing probe temperature decreases from 300.282 to 300.092 K. Considering both temperature-rise suppression and local ventilation capability, the 24 mm spacing scheme shows better radiation shielding and natural ventilation performance.
(3) The guide-plate inclination angle affects the airflow distribution near the probe. When the inclination angle is too small, the airflow-guiding effect is insufficient; when it is too large, local flow resistance may increase, and low-velocity regions may form inside the sensor. Considering both temperature-rise suppression and local ventilation, 45° can be selected as the preferred guide-plate inclination angle for the proposed structure.
(4) The multi-model comparison results show that, on the CFD simulation test dataset, the RMSE, MAE, and r of the MLP model are 0.0032 °C, 0.0020 °C, and 0.9979, respectively, outperforming those of the MLR and SVR models. This indicates that the MLP model can effectively describe the nonlinear dependence of radiation error on multiple environmental factors.
(5) Field comparative experiments show that the MLP model can predict radiation error with an RMSE of 0.052 °C, an MAE of 0.042 °C, and a correlation coefficient of 0.92. The obtained results suggest the effectiveness of the established correction method in improving the measurement performance of the naturally ventilated temperature sensor under real environmental conditions.

In summary, the proposed naturally ventilated air temperature sensor has a simple structure and requires no active ventilation, which indicates its potential advantages in reducing power consumption and maintenance requirements compared with aspirated shields. The field comparison experiment preliminarily demonstrates the effectiveness of the proposed sensor and correction method in reducing radiation-induced temperature errors under the tested outdoor conditions. These results indicate its potential for meteorological observation, air-quality monitoring, and agricultural microclimate assessment. Nevertheless, long-term field measurements are still required to quantitatively evaluate its actual power consumption, maintenance frequency, operational stability, and long-term robustness under different seasons, weather conditions, wind regimes, radiation levels, humidity ranges, land-cover surfaces, altitudes, and climatic regions. In addition, the response time of the proposed sensor was not independently tested in this study and will be evaluated under controlled dynamic conditions in future work.

## Figures and Tables

**Table 1:** Thermophysical properties of materials used in the CFD simulations.

Material	Density (kg·m^−3^)	Heat Capacity (J·kg^−1^·K^−1^)	Thermal Conductivity (W·m^−1^·K^−1^)
Aluminum	2719	871	202.4
Plastic	110	1591	0.2
Copper	8978	381	387.6

**Table 2:** Mesh independence test results based on probe temperature.

Mesh Level	Number of Cells	Probe Temperature (K)	Mesh Quality
Coarse mesh	427,939	300.094	≥0.25
Medium mesh	834,640	300.092	≥0.30
Fine mesh	2,581,741	300.092	≥0.30

**Table 3:** Simulation ranges for the investigated environmental variables.

Environmental Factor	Symbol	Default Value	Variation Range
Solar radiation	*P*_1_	1000	50–1200 W/m^2^
Long-wave radiation	*P*_2_	300	50–500 W/m^2^
Scattered radiation	*P*_3_	200	50–300 W/m^2^
Air density	*ρ*	1.225	0.7361–1.225 kg/m^3^
Ambient wind speed	*V*	1	0.5–8 m/s
Solar elevation angle	*E*	45°	10–90°
Surface albedo	*f*	0.2	0.1–0.9

**Table 4:** Comparison of prediction performance of different regression models on the test set.

Model	RMSE/°C	MAE/°C	r
MLR	0.0319	0.0220	0.7304
SVR	0.0098	0.0044	0.9798
MLP	0.0032	0.0020	0.9979

**Table 5:** Comparison of radiation-error statistics before and after correction.

Error Metric	Before Correction/°C	After Correction/°C
MBE	0.104	0.041
RMSE	0.127	0.052
MAE	0.104	0.042
Maximum absolute error	0.425	0.171
95th percentile absolute error	0.248	0.067

## Data Availability

The datasets generated and/or analyzed during the current study are available from the corresponding author on reasonable request.
